# Does Journal Content in the Field of Women's Health Represent Women's Burden of Disease? A Review of Publications in 2010 and 2020

**DOI:** 10.1089/jwh.2021.0425

**Published:** 2022-05-16

**Authors:** Laura Hallam, Amy Vassallo, Ana-Catarina Pinho-Gomes, Cheryl Carcel, Mark Woodward

**Affiliations:** ^1^ The George Institute for Global Health, University of New South Wales, Sydney, Australia.; ^2^ The George Institute for Global Health, School of Public Health, Imperial College London, London, United Kingdom.; ^3^ Sydney School of Public Health, Sydney Medical School, The University of Sydney, Sydney, Australia.

**Keywords:** women's health, global burden of disease, reproductive health, noncommunicable disease

## Abstract

**Background:**

Historically, women's health has focused on reproductive health. However, noncommunicable and communicable diseases comprise much of the burden of disease in women.

**Methods:**

A quantitative analysis of the main health content of articles published in six women's health journals (WHJ) and five general medical journals (GMJ) in 2010 and 2020 was conducted to categorize the main medical area topics of published articles and the life stage under study. Findings were compared with the leading causes of disease in women according to the Global Burden of Disease (GBD) study.

**Results:**

There were 1483 articles eligible for analysis. In total, in WHJ, 44% of topics were reproductive health, increasing from 36% in 2010 to 49% in 2020, which was similar to GMJ. Noncommunicable disease was the next most addressed topic, with cancer being the major disease area covered. When compared with the GBD study, major disease areas such as infectious disease, cardiovascular disease, and musculoskeletal disorders were underrepresented as topics in women's health publications. Most articles that focused on a particular life stage were on pregnancy or the reproductive years, with very few articles on menopause.

**Conclusion:**

Women's health publishing remains largely focused on reproductive health topics, with few articles on many of the major causes of morbidity and mortality in women. Journals, researchers, funders, and research priority setters should embrace a broader view of women's health to effectively cover content that reflects the broad range of health issues impacting women across the life span.

## Introduction

Reproductive health has historically been the center of focus for women's health by the medical establishment, applying what has been coined as the “bikini approach” to medicine.^1^ This limited view of women's health, while covering important issues, excludes many of the main causes of mortality and morbidity for women, as well as the broad range of issues impacting women across the life span and around the globe. While improving maternal and infant mortality was a substantial public health challenge in the 20th century, the global burden of disease (GBD) has changed significantly in recent years.

Noncommunicable diseases (NCDs) are now the leading cause of death and disability for women in most countries, with a high burden of disease in low- and middle-income countries.^2^ Cardiovascular disease, neoplasms, and chronic respiratory disease were the top three causes of death in women of all ages in 2019, according to the GBD study.^3^ Other leading conditions contributing to mortality and morbidity include musculoskeletal conditions, mental disorders, respiratory infections, and neurological disorders, as well as neonatal conditions.^3^ While many of these widespread NCDs are not unique to women, evidence has been increasingly demonstrating that sex and gender have a significant impact on disease presentation, outcomes, experiences of care, and exposure to risk factors.^1,2,4,5^

Thus, studying these diseases with attention to sex and gender provides essential insight that benefits women and assists in positioning these conditions as a women's health issue.

Women's health publications are a vital element in reshaping the field of women's health to cover the broad range of issues impacting women across the life span. A content analysis performed by Clark et al.^6^ in 2002 of women's health content in general medical and women's health journals (WHJ) found that while women's health specialty journals contained more nonreproductive content than women's health articles in general medical journals (GMJ), neither contained strong coverage of the leading causes of morbidity and mortality in women. Examining trends in publishing gives an indication of how the research focus in the field of women's health is evolving in the face of societal changes, at least in many high-income countries, providing insight into the impact of wider funding and strategic decisions on women's health research and prioritization.

We thus conducted an analysis of women's health articles published in six WHJ and five GMJ over the last 10 years. We aimed to determine whether their content covered the broad range of health issues impacting women across the life span, and if the major causes of morbidity and mortality were featured alongside reproductive health issues, and whether the coverage has changed over time. In addition, we aimed to assess if content on nonsex-specific diseases contained a sex and/or gender disaggregated analysis as this is essential to provide a controlled context to any findings for women.

## Materials and Methods

We conducted a quantitative analysis of the main health content of research articles published in WHJ and women's health-focused articles in GMJ in 2020, the most recent full year of publications, and 2010, the approximate midpoint from Clark et al's^6^ analysis. To select the WHJ, PubMed's journal list was searched with the terms “women” and “health.” Journals were included if they covered women's health without focus on any particular topic or discipline, contained “women” and “health” in the title, and were fully indexed in PubMed and Scopus.

**FIG. 1.**
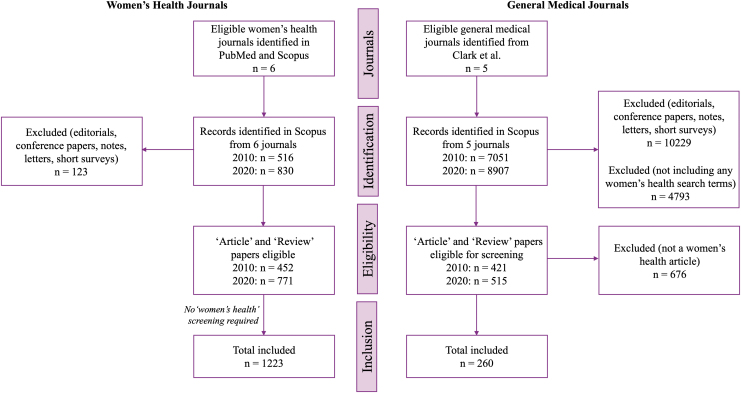
Flow diagram demonstrating the selection of journals and eligible articles from both WHJ and GMJ. GMJ, general medical journals; WHJ, women's health journals.

Eligible journals were *Journal of Women's Health*, *Women's Health*, *BMC Women's Health*, *Women's Health Issues*, *International Journal of Women's Health*, and *Women and Health.* The GMJ selected were those previously analyzed by Clark et al.,^6^ which were *Annals of Internal Medicine*, the *British Medical Journal* (BMJ), the *Journal of the American Medical Association* (JAMA), the *Lancet*, and *New England Journal of Medicine* (NEJM). Articles published in 2010 or 2020 and categorized as an “Article” or “Review” by Scopus were exported and considered eligible for analysis (Fig. 1). Editorials, notes, letters, and conference articles were ineligible.

For the articles from GMJ, further eligibility criteria were needed to identify women's health-focused articles. Titles and abstracts were additionally searched for mentions of women/females/girls, sex, gender, and specific health and anatomical terms (full list of terms in Supplementary Appendix SA1). The identified articles were screened for eligibility by two researchers and included if they were on (1) a women-specific issue, (2) studied only women as participants, (3) had a focus on women's health, or (4) aimed to conduct analysis by sex and/or gender as demonstrated by the objective, results, and conclusion of the abstract.

Data extraction was based on the title and abstract. Clinical experts in the authorship team created a list of topics based on medical/disease area, which were grouped into five high-level topic areas adapted from GBD categorizations: reproductive health, NCD, communicable disease, injury, and other (Table 1).^3^ Each article was classified in up to two topics and those addressing more than two main topics or not about a major medical discipline were categorized as “Other.” Subcategories of “Other,” added as the review was underway, included “Lifestyle and Risk Factors” and “Career,” as these were repeat topics.

**Table 1.** Main Medical Topic Area for Coding and Organization into High-Level Categories

High-level topic area	Topics
Reproductive health	Obstetrics Gynecology Sexual and reproductive health
Noncommunicable disease	Cardiovascular Metabolism and endocrinology Neurology Urology Digestive and nutrition Respiratory Cancer Mental illness and substance abuse Musculoskeletal Hematology Rheumatology Dermatology
Communicable disease	Infectious disease HIV
Injury	Violence and intentional injury Unintentional injury
Other	Lifestyle and risk factors Career Other

Articles were classified into the following life stages based on the terminology or age ranges provided by the authors: childhood (<10 years old), adolescence (10–19 years), reproductive years (15–50 years), pregnancy, menopause (45–55 years,^7^ about the menopausal period), midlife (45–60 years, not specifically about menopause), and postmenopausal (>55 years). If a topic was exclusively relevant to one group, such as gestational diabetes, the article was classified in that life stage. If articles covered multiple age groups or this was not specified in the abstract, they were categorized as such. Articles were recorded as focusing on a sex and/or gender analysis if they included men and women and analyzed data by sex and/or gender, and this was made clear in the abstract. Data were also extracted on the country of affiliation for the corresponding author of each article.

The coding framework was developed by all the authors in consultation. One author conducted data extraction on all the articles. Ten percent of the articles were selected for validation by coauthors, with three authors each reviewing an equal number of articles. Discrepancies were determined by consensus, with a fifth author consulting on the final decision where required. Any changes from the original coding were incorporated into the coding framework and applied to other relevant articles.

Descriptive statistics were used to summarize the proportions of high-level topic area, individual topics, life stage, and frequency of sex and/or gender analysis across all journals. The proportions of high-level topic area and life stages in the 2 years under study were compared using Pearson's chi-squared test. All analyses were undertaken in Excel, and statistical significance was taken as *p* < 0.05.

Results were compared with the GBD study's^3^ ranked data on disease burden, measured in disability adjusted life years (DALYs), and deaths caused by different disease categories for women. GBD data from 2009 to 2019 were used, as 2019 is currently the last available year.

This study did not require ethics approval.

## Results

Based on the eligibility criteria, 1223 articles from six WHJ and 260 articles from five GMJ were identified. In the WHJ, there were 452 articles in 2010, compared with 771 in 2020. In GMJ, there were 165 articles in 2010 and 95 in 2020, demonstrating a decrease in women's health content (Table 2).

**Table 2.** Number of Eligible Articles in Each Women's Health Journal and General Medical Journal for 2010 and 2020

Women's Health Journal	2010 (*n*)	2020 (*n*)	General Medical Journal	2010 (*n*)	2020 (*n*)
*BMC Women's Health*	35	264	*Annals of Internal Medicine*	8	16
*International Journal of Women's Health*	47	130	*British Medical Journal (BMJ)*	45	29
*Journal of Women's Health*	205	177	*Journal of the American Medical Association (JAMA)*	21	12
*Women and Health*	47	94	*Lancet*	36	16
*Women's Health*	56	46	*New England Journal of Medicine (NEJM)*	55	22
*Women's Health Issues*	62	60			
*Total*	*452*	*771*	*Total*	*165*	*95*

### High-level topic comparisons of 2010 and 2020

**FIG. 2.**
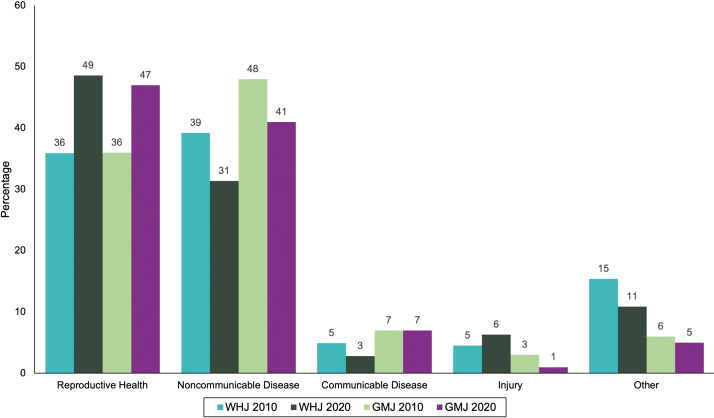
Percentage of topics in each high-level topic area in 2010 and 2020 across all six WHJ and all five GMJ.

The proportional coverage of each topic, relative to the total number of topics covered by the articles, was determined and compared between the 2 years (Fig. 2). In 2010, NCD was the most common high-level topic area, comprising 39.2% of topics in WHJ and 47.6% in GMJ, followed by reproductive health with 35.9% of topics in WHJ and 35.8% in GMJ. In 2020, this was reversed, with reproductive health having the most topics at 48.6% and 46.8% compared with 31.4% and 40.5% for NCD in WHJ and GMJ, respectively. There were very few articles covering communicable disease and injury. There was a significant association between publication year and topic area covered for the WHJ (*p* = 0.00002), but not for the GMJ (*p* = 0.3).

### Individual-level topic comparisons of 2010 and 2020

**FIG. 3.**
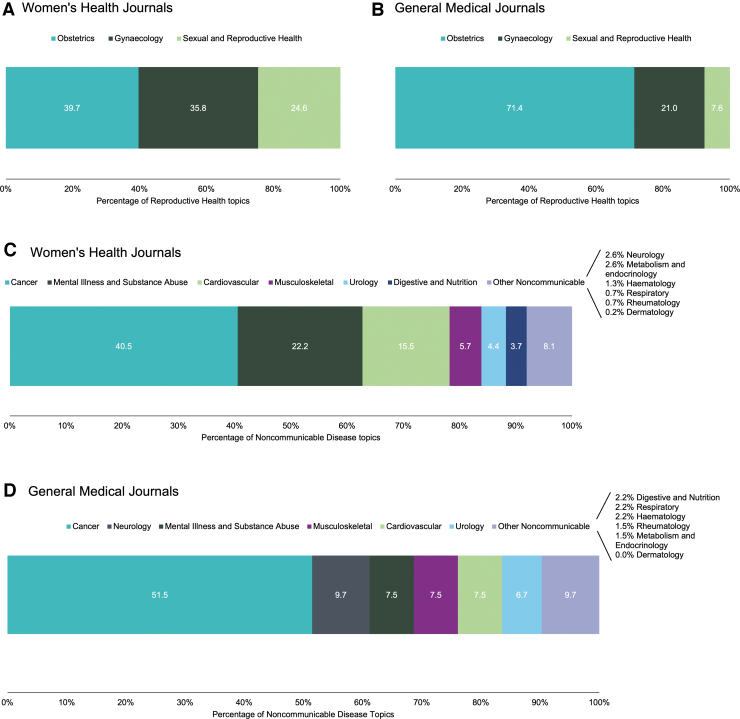
**(A)** Percentage of individual topics comprising the reproductive health topic area in WHJ. **(B)** Percentage of individual topics comprising the reproductive health topic area in GMJ. **(C)** Percentage of individual topics comprising the NCD topic area in WHJ. **(D)** Percentage of individual topics comprising the NCD topic area in GMJ. 2010 and 2020 data combined. Topics are presented *left* to *right* in the same order as the graph legends. NCD, noncommunicable disease.

Further breakdown of the high-level topic area to individual topics showed similar trends between 2010 and 2020 (Supplementary Tables S1 and S2) and thus a combined analysis was undertaken. For the WHJ, reproductive health topics were evenly distributed between obstetrics and gynecology (39.7% and 35.8%, respectively), with 24.6% on sexual and reproductive health (Fig. 3A). In the GMJ, obstetrics dominated with 71.4% of topics and 7.6% were on sexual health (Fig. 3B).

Cancer was by far the most covered NCD topic in WHJ (Fig. 3C), at 40.5%, followed by mental illness and substance abuse at 22.2%. Cardiovascular disease made up 15.5% of NCD articles. Musculoskeletal disorders (5.7%), urology (4.4%), and digestive and nutrition (3.7%) were less commonly covered. Topics with <3% of the total were combined as “other noncommunicable,” including neurology (2.6%), metabolism and endocrinology (2.6%), respiratory (0.7%), hematology (1.3%), rheumatology (0.7%), and dermatology (0.2%), which all had extremely low coverage despite including many major diseases. For the GMJ, 51.5% of NCD topics were on cancer (Fig. 3D), followed by neurology (9.7%), mental illness, substance abuse, and musculoskeletal and cardiovascular (all 7.5%), and urology (6.7%), with all others having <3%.

### Life stage

**FIG. 4.**
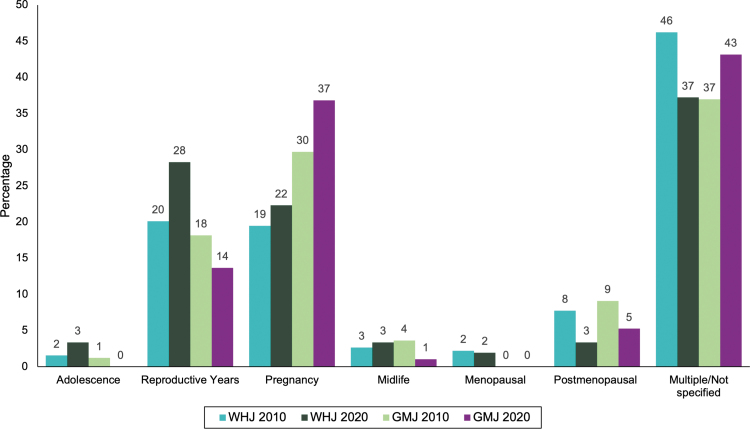
Percentage of articles specific to each life stage in 2010 and 2020 in all six WHJ and all five GMJ, excluding three articles on childhood.

The articles were also categorized according to life stage (Fig. 4). The majority of articles across both sets of journals covered multiple life stages or did not identify any specific life stage. In the WHJ, the remainder of the articles were largely focused on the reproductive years (20.1% in 2010 and 28.3% in 2020) and pregnancy (19.5% in 2010 and 22.3% in 2020). Only one article was on childhood. There was again a significant association between publication year and life stage studied for the WHJ (*p* = 0.0005). For the GMJ, pregnancy dominated (29.7% in 2010 and 36.8% in 2020), while there were 18.2% and 13.7% on the reproductive years in 2010 and 2020, respectively. All other life stages had fewer than 10% of the articles addressing their populations, with no GMJ articles across either year exclusively studying menopausal women.

### Sex and gender

Few articles focused on a sex and/or gender-based analysis, as defined by the authors. Only a small number of articles fit into this category, with 8.4% and 7.3% in 2010 and 4.8% and 7.4% in 2020 for the WHJ and GMJ, respectively.

### Country of affiliation

Articles in the WHJ had corresponding authors from 77 different countries over the 2 years, while the GMJ spanned 28 countries (Supplementary Tables S3 and S4). However, 49% of total articles were from the United States for both WHJ and GMJ. In the WHJ, there was a large increase in articles from the Asia Pacific, North Africa and the Middle East and sub-Saharan Africa from 2010 to 2020, contributing to much of the increase in total article numbers as the number from North America stayed stable. Overall, North America had the lowest percentage of reproductive health topics (37%) and sub-Saharan Africa the highest (64%), while South America had the highest proportion on NCD (42%). In the GMJ, the vast majority of articles were from North America and Europe.

### Comparison between topics of articles and causes of disease burden

**FIG. 5.**
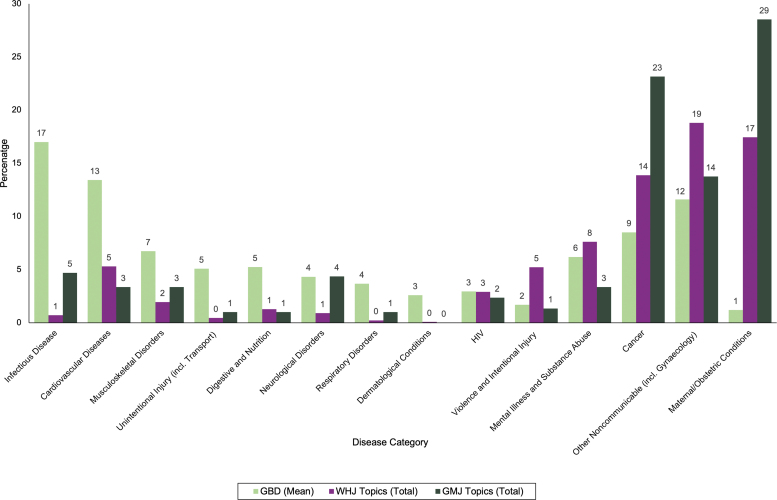
Comparison of the proportion of DALYs attributable to each disease category based on the mean of GBD data from 2009 to 2019, with the proportion of topics in each disease category in all six WHJ and all five GMJ across both 2010 and 2020. DALYs, disability adjusted life years; GBD, Global Burden of Disease.

There were stark differences between the article topics covered and their impact on women's morbidity and mortality. Reproductive health was significantly more prevalent as a topic in women's health publications compared with its disease burden (Fig. 5). Maternal conditions make up only 1.2% of DALYs, but accounted for 17.4% of topics in WHJ and 28.5% in GMJ. Gynecology was combined with other NCD in the GBD data, which when compared with the topic breakdown, led to the WHJ having a much higher proportion in this category. However, gynecological conditions make up only 2.3% of DALYs individually.

Conversely, cardiovascular disease makes up 13.4% of DALYs and only 5.3% of topics in WHJ and 3.4% in GMJ. Also, relatively underrepresented are musculoskeletal disorders, unintentional injury, digestive and nutrition, respiratory disorders, and dermatological conditions. The high coverage of predominantly breast and cervical cancers led to cancer comprising 13.9% of topics in WHJ and 23.2% in GMJ, compared with 8.5% of DALYs. Notably, infectious diseases account for 17.0% of DALYs, but only 0.7% of topics in WHJ and 4.7% in GMJ. Comparison with GBD mortality data showed broadly similar trends (Supplementary Table S5).

## Discussion

WHJ showed an overall increase in articles from 2010 to 2020 and a diversification of global authorship, while women's health content decreased in GMJ in the same period, with a mostly Western authorship.

Despite the emergence of a new women's health agenda advocating for a broader definition of women's health^2^ and increased focus on NCDs,^8^ our study has demonstrated that reproductive health topics comprise a large proportion of content in women's health publications, making up 44% of total women's health topics across 2010 and 2020 combined in WHJ and 40% in GMJ. Most notably, there was an increase in the proportion of reproductive health topics from 2010 to 2020, demonstrating an increased focus on reproductive health over time. GMJ articles on women's health had a dominant focus on obstetrics, and the NCD topics were mostly on cancer, demonstrating a lack of diversity in content.

These results largely mirror Clark et al.'s^6^ 2002 study, which showed that GMJ women's health content was more focused on reproductive health than WHJ, demonstrating a striking lack of change in 20 years. The overall focus on reproduction rather than engaging with a broader view of women's health may continue to reflect and perpetuate society's, scientists’, and clinicians' view of women's health as synonymous with reproductive health. Reproductive health was additionally focused on pregnancy and the reproductive years, with an extraordinarily low proportion of articles focused on menopause, only 2% in the WHJ and none in the GMJ.

There were very low proportions of articles focused on midlife and postmenopausal women, leaving a significant proportion of women and their specific health concerns underrepresented. Life expectancies are increasing and while women's life expectancies are generally longer than men's, women have fewer healthier years and high rates of disability in older age.^9^ Thus, it is important to examine women's health and well-being across the life span and study the impact of diseases that are prevalent in old age, which may disproportionately impact women.

The increase in reproductive health topics from 2010 to 2020 can potentially be explained by scientific priorities and policies from major global and national institutions. The introduction of the United Nations Sustainable Development Goals (SDGs) from 2015 reaffirmed the focus on maternal health of the previous Millennium Development Goals, with SDG 3 on Good Health and Well-Being having targets to improve maternal and neonatal mortality and to improve access to sexual and reproductive health care.^10^ The UN's Every Woman Every Child initiative, which focused on maternal and child health, was introduced in 2010 and firmly aligns with SDG 3.^11^

The observed concurrent increase in research emerging from lower income countries in 2020 may align with work targeting these SDGs. In addition, increased discourse about understudied, but extremely common, gynecological conditions such as endometriosis and polycystic ovarian syndrome may also have impacted an increase in focus on reproductive health.^12^ It is, of course, important to address the knowledge gaps surrounding these poorly understood and underdiagnosed diseases. However, these conditions comprised only a fraction of the articles on reproductive health.

Our comparison with the GBD data clearly shows major disease areas that comprise a significant burden to women globally and across the life span are not being covered in women's health publications, including NCDs such as cardiovascular disease, stroke, musculoskeletal, respiratory and neurological conditions, as well as infectious disease. Articles on autoimmune conditions were extremely limited, for example, only 0.4% of topics were classified as rheumatology, despite autoimmune disease disproportionately impacting women,^13^ although some autoimmune diseases have been classified under other medical areas they are relevant to.

Cancer was the main content area for most (40.5% in WHJ and 51.5% in GMJ) articles on NCDs, but these were dominated by breast and cervical cancers with very little on other major cancers burdening women, such as lung and colorectal cancer.^3^ Also, notably absent from the articles published in the WHJ in 2020 was coronavirus disease 2019 (COVID-19). Only 8 articles of the 771 published in 2020 were about the COVID-19 pandemic, despite significant research and discussion on the gendered impacts of the disease, including the burden on the predominantly women health care workforce, increased domestic load, lack of access to health care, and rising rates of domestic violence.^14–17^ Violence and intentional injury, including sexual violence, was also underaddressed, especially in the GMJ.

The absence of these topics from women's health publications may prevent high-burden, nonsex-specific conditions from being perceived as a women's health issue. However, conditions that are considered women's health issues have been defined since the 1980s as those that are unique to, more prevalent or more serious in women, or have risk factors or interventions that are different in women.^18^

An increased focus in recent years on the role of sex and gender in health and disease has demonstrated that many conditions fall under this definition of women's health. Sex and gender bias in research and health care can lead to poorer health outcomes for women, particularly in conditions not recognized as women's health issues.^19–22^ This reinforces the importance of analyzing sex and gender in health and medical research to have an appropriate knowledge base to understand the specific experiences of women.

A 2010 study showed that the quantity of sex- and gender-focused research varied by discipline, but was increasing.^23^ This movement toward sex- and gender-sensitive research has been gaining traction, with major international institutions introducing policies to promote or mandate the incorporation of sex and gender in research.^24^

The US National Institutes of Health (NIH) announced its new Sex As a Biological Variable policy in 2014,^25^ encouraging the consideration of the influence of sex in human and animal research, and a further call to action was published in 2020 due to limited progress.^26^ The Canadian Institutes of Health Research (CIHR) and the European Commission have also introduced policies and further requirements for considering sex and gender in research.^27^ The Sex and Gender Equity in Research (SAGER) publishing guidelines were also released between 2010 and 2020 and endorsed by many journals.^28^

Despite this, there were limited numbers of articles in both sets of journals reviewed here that focused on a sex and/or gender analysis and the disciplines that were highly represented in sex and gender research in general, according to Oertelt-Prigione et al.,^23^ such as cardiology and endocrinology were not well covered in women's health publications. This shift by major institutions may have cemented sex and gender research as more widely relevant, moving away from the roots of this field in the women's health movement and shifting sex- and gender-focused research out of WHJ, renewing a focus on reproductive health. It also may have led to sex- and gender-based analysis being incorporated into studies with wider aims, rather than researchers focusing on sex and gender impacts as the main aims and outcome of the article.

While examining publishing trends is an effective way to understand current research focuses, publishing is downstream of institutional decisions on research priorities and funding decisions. A 2021 study in the Journal of Women's Health showed that many conditions that predominately impact women were underfunded by the NIH compared with their impact on US populations, including rheumatoid arthritis, migraine, endometriosis, irritable bowel syndrome, multiple sclerosis, and myalgic encephalomyelitis.^29^

With half of all articles in our study emerging from the United States, the decisions of major funders such as the NIH have a significant impact on women's health literature. Thus, the responsibility to broaden women's health research and tackle underaddressed health conditions impacting women lies with many, including funders. Despite upstream factors influencing research priorities and funding, journal editors and reviewers can maintain awareness of the shifting disease burden in women and understand which conditions are understudied and promote this work, particularly in the high-impact GMJ. We recommend that editors make a conscious effort to monitor diversity of publications in their journals and the wider field to ensure inclusion of a breadth of women's health issues, and that reviewers are appropriately briefed on the scope of the journal.

Our study contains several limitations. The coding of articles into medical topic areas was based only on the title and abstract and was limited to two topics, which could have led to additional health focuses not being captured in the extraction, particularly if they addressed a more holistic view of health. In addition, the comparison between the topic areas and the GBD data is limited by the categorizations used in both studies, allowing only approximate equivalences. GBD data should be taken as an estimation of the total burden of disease globally and provides decontextualized information that does not capture many factors that influence health. Further insight on the change over time could have been gained by expanding the years under study.

Our assessment of whether a sex and/or gender analysis was undertaken, or was a main aim, was based only on the title and abstract, and so, we were unable to examine whether the principles of the SAGER guidelines were applied. We also acknowledge that many researchers are incorporating sex and/or gender disaggregated data and/or analysis into their work, which helps to build knowledge, even if that is not the main aim of their study. We have also only looked at women's health-specific and high-impact GMJ and acknowledge there will be research addressing NCD, communicable diseases, and injury in lower impact factor, discipline-specific or sex- and gender-focused journals.

We issue a call to action to research funders, publishers, and reviewers to broaden the focus of women's health and actively prioritize underresearched conditions as well as those comprising the greatest burden of disease in women globally, including NCD, communicable diseases, and injury. We recommend that WHJ and GMJ increase the representation of articles aimed at understanding the impacts of sex and gender on health, disease, and health care and recommend use of the SAGER guidelines when designing studies and reporting data on sex and gender.^28^

We also encourage the platforming of diverse scientific voices and examination of different women's unique experiences of disease across the globe. We recommend that researchers also embrace this wide-ranging view of women's health to assist in changing the scientific culture to view women's health needs beyond reproductive care.

## Conclusion

We conducted a thorough analysis of women's health content in both WHJ and high-impact GMJ and how it has changed in the last decade, providing up-to-date information on the current breadth of dedicated and influential women's health literature. Women's health publications are dominated by reproductive health topics, despite calls for a broadening view of women's health and the incorporation of sex and gender into the study of nonsex-specific health conditions. Many diseases that contribute to major morbidity and mortality in women such as cardiovascular disease, stroke, and chronic lung diseases were poorly covered in women's health publications. This is despite journals' invitations for articles examining a broad definition of women's health across the life span.

We recommend that journals, funders, and researchers need to work to broaden societal and scientific understanding of women's health, ensuring that women of all ages are able to be appropriately and effectively served by scientific research and health care.

## Supplementary Material

Supplemental data

Supplemental data

Supplemental data

Supplemental data

Supplemental data

Supplemental data

## Supplementary Material

Supplementary Appendix SA1
