# Supplementary material for: Does Journal Content in the Field of Women's Health Represent Women's Burden of Disease? A Review of Publications in 2010 and 2020
Source: J Womens Health (Larchmt). 2022 May 16;31(5):611–9. doi: 10.1089/jwh.2021.0425 (PMC9133969; doi:10.1089/jwh.2021.0425)
Supplement: Supplemental data [file Suppl_TableS1.docx]

*Table S1. Proportion of reproductive health topics in each individual topic area by year for both A) women’s health journals and B) general medical journals*

| ***A) Women’s Health Journals*** | | | |
| --- | --- | --- | --- |
| **Year** | **Obstetrics** | **Gynaecology** | **Sexual and Reproductive Health** |
| 2010 | 39.4 | 36.6 | 24.0 |
| 2020 | 39.8 | 35.4 | 24.8 |
| ***B) General Medical Journals*** | | | |
| **Year** | **Obstetrics** | **Gynaecology** | **Sexual and Reproductive Health** |
| 2010 | 73.1 | 16.4 | 10.4 |
| 2020 | 69.2 | 26.9 | 3.8 |
