# Supplementary material for: Does Journal Content in the Field of Women's Health Represent Women's Burden of Disease? A Review of Publications in 2010 and 2020
Source: J Womens Health (Larchmt). 2022 May 16;31(5):611–9. doi: 10.1089/jwh.2021.0425 (PMC9133969; doi:10.1089/jwh.2021.0425)
Supplement: Supplemental data [file Suppl_TableS2.docx]

*Table S2. Proportion of non-communicable disease topics in each individual topic area by year for both A) women’s health journals and B) general medical journals. Individual topic areas with ≤ 3% of topics were combined in ‘Other’.*

| ***A) Women’s Health Journals*** | | | | | | | |
| --- | --- | --- | --- | --- | --- | --- | --- |
| **Year** | **Cancer** | **Mental Illness and Substance Abuse** | **Cardiovascular** | **Musculoskeletal** | **Urology** | **Digestive and Nutrition** | **Other** |
| 2010 | 39.8 | 16.2 | 16.8 | 8.9 | 3.7 | 3.7 | 11.0 |
| 2020 | 41.0 | 26.5 | 14.6 | 3.4 | 4.9 | 3.7 | 6.0 |
| ***B) General Medical Journals*** | | | | | | | |
| **Year** | **Cancer** | **Neurology** | **Mental Illness and Substance Abuse** | **Musculoskeletal** | **Cardiovascular** | **Urology** | **Other** |
| 2010 | 46.1 | 12.4 | 7.9 | 9.0 | 7.9 | 6.7 | 10.1 |
| 2020 | 62.2 | 4.4 | 6.7 | 4.4 | 6.7 | 6.7 | 8.9 |
