# Supplementary material for: Does Journal Content in the Field of Women's Health Represent Women's Burden of Disease? A Review of Publications in 2010 and 2020
Source: J Womens Health (Larchmt). 2022 May 16;31(5):611–9. doi: 10.1089/jwh.2021.0425 (PMC9133969; doi:10.1089/jwh.2021.0425)
Supplement: Supplemental data [file Suppl_TableS3.docx]

*Table S3. Number of articles in A) women’s health journals and B) general medical journals from each world region per year, based on corresponding author affiliation*

| ***A) Women’s Health Journals*** | | | |
| --- | --- | --- | --- |
| **Region** | **2010** | **2020** | **Total Articles** |
| Europe | 72 | 100 | 172 |
| North America | 326 | 326 | 652 |
| South America | 5 | 18 | 23 |
| Asia-Pacific | 31 | 134 | 165 |
| North Africa and Middle East | 9 | 86 | 95 |
| Sub-Saharan Africa | 9 | 107 | 116 |
| *Total* | *452* | *771* | *1223* |
| ***B) General Medical Journals*** | | | |
| **Region** | **2010** | **2020** | **Total Articles** |
| Europe | 62 | 36 | 98 |
| North America | 87 | 55 | 142 |
| South America | 1 | 0 | 1 |
| Asia-Pacific | 12 | 2 | 14 |
| North Africa and Middle East | 1 | 1 | 2 |
| Sub-Saharan Africa | 2 | 1 | 3 |
| *Total* | *165* | *95* | *260* |
