# Supplementary material for: Does Journal Content in the Field of Women's Health Represent Women's Burden of Disease? A Review of Publications in 2010 and 2020
Source: J Womens Health (Larchmt). 2022 May 16;31(5):611–9. doi: 10.1089/jwh.2021.0425 (PMC9133969; doi:10.1089/jwh.2021.0425)
Supplement: Supplemental data [file Suppl_TableS4.docx]

*Table S4. Proportion of topics in A) women’s health journals and B) general medical journals in each high-level category, by region*

| ***A) Women’s Health Journals*** | | | | | |
| --- | --- | --- | --- | --- | --- |
| **Region** | **Reproductive Health** | **Non-Communicable Disease** | **Communicable Disease** | **Injury** | **Other** |
| Europe | 45% | 37% | 2% | 5% | 11% |
| North America | 37% | 37% | 5% | 6% | 15% |
| South America | 58% | 42% | 0% | 0% | 0% |
| Asia-Pacific | 47% | 36% | 1% | 5% | 10% |
| North Africa and Middle East | 60% | 23% | 2% | 4% | 11% |
| Sub-Saharan Africa | 64% | 19% | 5% | 7% | 6% |
| ***B) General Medical Journals*** | | | | | |
| **Region** | **Reproductive Health** | **Non-Communicable Disease** | **Communicable Disease** | **Injury** | **Other** |
| Europe | 47% | 44% | 5% | 1% | 3% |
| North America | 36% | 49% | 6% | 1% | 8% |
| South America | 0% | 50% | 0% | 50% | 0% |
| Asia-Pacific | 37% | 37% | 11% | 11% | 5% |
| North Africa and Middle East | 50% | 0% | 50% | 0% | 0% |
