# Supplementary material for: Does Journal Content in the Field of Women's Health Represent Women's Burden of Disease? A Review of Publications in 2010 and 2020
Source: J Womens Health (Larchmt). 2022 May 16;31(5):611–9. doi: 10.1089/jwh.2021.0425 (PMC9133969; doi:10.1089/jwh.2021.0425)
Supplement: Supplemental data [file Suppl_TableS5.docx]

*Table S5. Comparison between mean GBD statistics for 2009 and 2019 and proportion of total women’s health topics covered in women’s health and general medical journals for 2010 and 2020 for major disease areas*

| **Topics** | **GBD**  **(Mean % of DALYs)** | **GBD**  **(Mean % of Deaths)** | **Women’s Health Journals**  **(% of Topics)** | **General Medical Journals**  **(% of Topics)** |
| --- | --- | --- | --- | --- |
| Infectious disease | 17.0 | 14.4 | 0.7 | 4.7 |
| Cardiovascular Diseases | 13.4 | 33.1 | 5.3 | 3.4 |
| Musculoskeletal Disorders | 6.7 | 0.3 | 1.9 | 3.4 |
| Unintentional Injury (Incl. Transport) | 5.1 | 4.0 | 0.4 | 1.0 |
| Digestive and Nutrition | 5.2 | 4.5 | 1.3 | 1.0 |
| Neurological Disorders | 4.3 | 4.6 | 0.9 | 4.4 |
| Respiratory Disorders | 3.7 | 6.5 | 0.2 | 1.0 |
| Dermatological Conditions | 2.6 | 0.2 | 0.1 | 0.0 |
| HIV | 2.9 | 2.5 | 2.9 | 2.3 |
| Violence and Intentional Injury | 1.7 | 1.4 | 5.2 | 1.3 |
| Mental Illness and Substance Abuse | 6.2 | 0.2 | 7.6 | 3.4 |
| Cancer | 8.5 | 15.9 | 13.9 | 23.2 |
| Other Non-Communicable (incl. Gynaecology | 11.6 | 7.4 | 18.8 | 13.8 |
| Maternal/Obstetric Conditions | 1.2 | 0.9 | 17.4 | 28.5 |
