# Supplementary material for: Does Journal Content in the Field of Women's Health Represent Women's Burden of Disease? A Review of Publications in 2010 and 2020
Source: J Womens Health (Larchmt). 2022 May 16;31(5):611–9. doi: 10.1089/jwh.2021.0425 (PMC9133969; doi:10.1089/jwh.2021.0425)
Supplement: Supplemental data [file Suppl_AppSA1.docx]

**Supplementary**

*Appendix 1. Search terms used to identify articles in general medical journals related to women’s health using Scopus.*

women OR woman OR female* OR girl* OR sex OR gender OR mother OR maternal OR maternity OR lesbian OR breast* OR ovary OR ovarian OR cervix OR cervical OR vagin* OR pregnan* OR prenatal OR antenatal OR perinatal OR postnatal OR postpartum OR pre-natal OR ante-natal OR peri-natal OR post-natal OR post-partum OR birth OR childbirth OR uterus OR uterine OR endometri* OR gynaecolo* OR obstetric* OR fallopian OR menopaus* OR menstrua* OR ovulat* OR abortion OR gestat* OR miscarriage OR vulva* OR hysterectomy OR oophorectomy OR salpingectomy OR "pelvic pain" OR oocyte OR ovum OR follicle OR oestrogen OR estrogen OR progesterone OR fertil* OR "hormone replacement therapy" OR reproduct*
